# Supplementary material for: Pyruvate Dehydrogenase Kinase 1 inhibition mediated oxidative phosphorylation enhancement in cartilage promotes osteoarthritis progression
Source: BMC Musculoskelet Disord. 2023 Jul 20;24:597. doi: 10.1186/s12891-023-06585-6 (PMC10357736; doi:10.1186/s12891-023-06585-6)
Supplement: Supplementary file 1 — Additional file 1. [file 12891_2023_6585_MOESM1_ESM.zip › BP.Gsea.1653623667859/GOBP_LATE_ENDOSOME_TO_VACUOLE_TRANSPORT.html]

Details for gene set GOBP\_LATE\_ENDOSOME\_TO\_VACUOLE\_TRANSPORT[GSEA]

|  || Dataset | 55457\_collapsed\_to\_symbols.cls.cls #OA\_versus\_normal.cls.cls #OA\_versus\_normal\_repos |
| Phenotype | cls.cls#OA\_versus\_normal\_repos |
| Upregulated in class | OA |
| GeneSet | GOBP\_LATE\_ENDOSOME\_TO\_VACUOLE\_TRANSPORT |
| Enrichment Score (ES) | 0.60946685 |
| Normalized Enrichment Score (NES) | 1.9105874 |
| Nominal p-value | 0.0021551724 |
| FDR q-value | 0.041899413 |
| FWER p-Value | 0.721 |
Table: GSEA Results Summary

  

Fig 1: Enrichment plot: GOBP\_LATE\_ENDOSOME\_TO\_VACUOLE\_TRANSPORT      
 Profile of the Running ES Score & Positions of GeneSet Members on the Rank Ordered List

  

| SYMBOL | TITLE | RANK IN GENE LIST | RANK METRIC SCORE | RUNNING ES | CORE ENRICHMENT || 1 | CHMP2B | charged multivesicular body protein 2B [Source:HGNC Symbol;Acc:HGNC:24537] | 129 | 0.876 | 0.1087 | Yes |
| 2 | LEPROTL1 | leptin receptor overlapping transcript like 1 [Source:HGNC Symbol;Acc:HGNC:6555] | 434 | 0.691 | 0.1788 | Yes |
| 3 | CHMP4A | charged multivesicular body protein 4A [Source:HGNC Symbol;Acc:HGNC:20274] | 526 | 0.649 | 0.2597 | Yes |
| 4 | CHMP5 | charged multivesicular body protein 5 [Source:HGNC Symbol;Acc:HGNC:26942] | 556 | 0.638 | 0.3439 | Yes |
| 5 | TMEM50A | transmembrane protein 50A [Source:HGNC Symbol;Acc:HGNC:30590] | 576 | 0.631 | 0.4279 | Yes |
| 6 | TMEM50B | transmembrane protein 50B [Source:HGNC Symbol;Acc:HGNC:1280] | 883 | 0.547 | 0.4784 | Yes |
| 7 | CHMP2A | charged multivesicular body protein 2A [Source:HGNC Symbol;Acc:HGNC:30216] | 1269 | 0.464 | 0.5114 | Yes |
| 8 | PIK3R4 | phosphoinositide-3-kinase regulatory subunit 4 [Source:HGNC Symbol;Acc:HGNC:8982] | 1424 | 0.439 | 0.5590 | Yes |
| 9 | VPS28 | VPS28 subunit of ESCRT-I [Source:HGNC Symbol;Acc:HGNC:18178] | 1522 | 0.426 | 0.6092 | Yes |
| 10 | CHMP6 | charged multivesicular body protein 6 [Source:HGNC Symbol;Acc:HGNC:25675] | 2140 | 0.345 | 0.6081 | Yes |
| 11 | CHMP3 | charged multivesicular body protein 3 [Source:HGNC Symbol;Acc:HGNC:29865] | 2733 | 0.277 | 0.5996 | Yes |
| 12 | SNF8 | SNF8 subunit of ESCRT-II [Source:HGNC Symbol;Acc:HGNC:17028] | 3037 | 0.246 | 0.6095 | Yes |
| 13 | LEPROT | leptin receptor overlapping transcript [Source:HGNC Symbol;Acc:HGNC:29477] | 5608 | 0.023 | 0.4131 | No |
| 14 | STAM | signal transducing adaptor molecule [Source:HGNC Symbol;Acc:HGNC:11357] | 6403 | -0.035 | 0.3563 | No |
| 15 | CHMP1B | charged multivesicular body protein 1B [Source:HGNC Symbol;Acc:HGNC:24287] | 7339 | -0.105 | 0.2980 | No |
| 16 | BECN1 | beclin 1 [Source:HGNC Symbol;Acc:HGNC:1034] | 8644 | -0.204 | 0.2244 | No |
| 17 | CHMP7 | charged multivesicular body protein 7 [Source:HGNC Symbol;Acc:HGNC:28439] | 9723 | -0.294 | 0.1805 | No |
| 18 | CHMP1A | charged multivesicular body protein 1A [Source:HGNC Symbol;Acc:HGNC:8740] | 11401 | -0.487 | 0.1164 | No |
Table: GSEA details [plain text format]

  

Fig 2: GOBP\_LATE\_ENDOSOME\_TO\_VACUOLE\_TRANSPORT      
 Blue-Pink O' Gram in the Space of the Analyzed GeneSet

  

Fig 3: GOBP\_LATE\_ENDOSOME\_TO\_VACUOLE\_TRANSPORT: Random ES distribution      
 Gene set null distribution of ES for **GOBP\_LATE\_ENDOSOME\_TO\_VACUOLE\_TRANSPORT**
